# A Tale of Two Cities: Reflections on Digital Technology and the Natural Environment

**DOI:** 10.1016/j.patter.2020.100068

**Published:** 2020-08-14

**Authors:** Gordon S. Blair

**Affiliations:** 1 School of Computing and Communications, Lancaster University, UK

## Abstract

Contemporary digital technologies can make a profound impact on our understanding of the natural environment in moving toward sustainable futures. Examples of such technologies included sources of new data (e.g., an environmental Internet of Things), the ability to storage and process the large datasets that will result from this (e.g., through cloud computing), and the potential of data science and AI to make sense of these data alongside human experts. However, these same trends pose a threat to sustainable futures through, for example, the carbon footprint of digital technology and the risks of this escalating through the very trends mentioned above.

## Main Text

### Introduction

I have a curious relationship with digital technology. I am enthusiastic about computer technology, having been involved in computer science research since the early ‘80s. On the other hand, I am deeply concerned about the impact that digital technology has on society. When I think about this, I often find myself drawn to the marvelous words of Charles Dickens—his opening lines of a *Tale of Two Cities* (written in 1859):

“It was the best of times, it was the worst of times, it was the age of wisdom, it was the age of foolishness, it was the epoch of belief, it was the epoch of incredulity, it was the season of Light, it was the season of Darkness, it was the spring of hope, it was the winter of despair, we had everything before us, we had nothing before us, we were all going direct to Heaven, we were all going direct the other way…”

In this short opinion piece, I reflect more on this “tale of two cities” with particular reference on the impact of digital technology on the natural environment, drawing on my experiences as in my Engineering and Physical Sciences Research Council (EPSRC) Senior Fellowship, awarded in 2016, and examining the role of digital technology in understanding and managing environmental change.

### Wisdom, Belief, and Light

Starting on the positive side, I am a passionate and firm advocate for how digital technology can help us to better understand the natural environment. During my fellowship, the team has worked with a range of environmental scientists on problems as diverse as supporting a more data-driven approach to flood risk management, understanding the complexities associated with biodiversity loss, and seeking better modeling paradigms for understanding soils. Let us unpick one of these pieces of work—on flood risk management.

Increased flooding is one of the most destructive consequences of climate change. Locally for us, flooding associated with Storm Desmond hit Lancashire and Cumbria hard and left 50,000 people without power, and the financial cost in Cumbria alone was estimated at £500 million. On a global scale, as reported by the United Nations (UN) in 2015, flooding impacted 2.3 billion people over a 20-year period, and 157,000 people have died.^1^ The challenges facing flood risk management practitioners are considerable as they make long-term decisions, e.g., about investments in flood defenses, with limited budgets. They have to deal with high levels of complexity and uncertainty and also increases in extreme events related to climate change. Traditionally, they would use a range of modeling tools for future projections, drawing on the rich tradition in process modeling in the area including global climate models, more local weather models, models related to catchment hydrology and inundation, and so on. Thanks to developments in digital technology, though, major changes are now anticipated, in particular related to the plethora of data becoming available (cf. big data)—from satellite imagery, from sensors deployed around catchments (cf. the Internet of Things), from detailed studies carried out by local authorities, from citizen science, and from mining data from the web. However, this is not without its challenges. We are moving toward having unprecedented amounts of data, but these data are highly heterogeneous and at different scales and accuracies. There is a danger that scientists and decision-makers may drown in this sea of data, and tools are urgently required to make sense of these complex data.^2^ Importantly, there is also a critical need to integrate understanding from process models with insights and trends emerging from the data. At present, there is good understanding of process modeling albeit with significant open research questions, for example, in terms of integrated modeling across multiple processes or in terms of managing uncertainty in process modeling. There are also significant advances in understanding data, especially with recent research in the fields of data science and artificial intelligence (AI). The real gap is how these two perspectives interact and inform each other.

In the fellowship work, we looked at how digital technology can support a more data-driven approach to flood risk management. The key innovation was a cloud-based data hypercube, which achieved the desired level of integration of highly heterogeneous data from a variety of sources. This hypercube was implemented using semantic web technologies particularly related to linked data with the hypercube also used to store process model output, allowing integrated analyses across process models and other forms of data. We also investigated the use of data mining techniques based on machine learning to enhance the information available in the hypercube. Notebook technologies (cf. Jupityr notebooks) were then used to provide collaborative access to analyses and investigations of future scenarios.

Looking more generally across our work, we claim digital technology can have a transformative impact on environmental sciences:

1 Digital technology can provide unprecedented levels of data for environmental scientists to work with, including real-time streaming data and a spatial and temporal resolution unimaginable a few years ago.
2 Cloud computing has the (elastic) capacity to store and process the resultant massive datasets. As a team, we are also particularly excited by the concept of virtual labs that offer collaborative access to environmental data and analytics capability in the cloud and that offer a paradigm shift toward a more open and integrative style of environmental science.
3 Data science is providing a range of innovative techniques to make sense of large datasets, with increasing focus on tailored techniques to address the particular challenges of environmental data, e.g., reasoning across scale, managing extremes, and integration with process understanding.^2^
4 Digital technology is also opening the way to new ways of communication, whether supporting decision-makers or reaching out to children to inspire them to become digital naturalists.^3^

### Foolishness, Incredulity, and Darkness

So, what could possibly be bad? Recently, I had the opportunity to work on a project looking at the climate impacts of digital technology in terms of current and future carbon emissions from the sector. A significant number of studies have been carried out, most notably by Malmodin and Lundén,^4^ Andrae and Edler,^5^ and Belkhir and Elmeligi.^6^ The headline figure is that the current share of greenhouse gas (GHG) emissions of ICT (Information and Communications Technology) is estimated to be between 1.8%–2.8% of global emissions. However, there is no clear agreement on the figures. Digging deeper the studies vary in (1) scope in that they all consider ICT as consisting of devices, communication networks, and data centers, but some omit important areas such blockchain or TV; and (2) the extent to which they consider the full supply chain and life cycles of different technologies. We estimate that this means the true carbon footprint of ICT is underestimated by 25% and runs at somewhere between 2.1% and 3.9% of global emissions. To put these numbers in context, the equivalent figure for the airline industry is around 2.5% (fuel only, although this is the dominant factor and ignores the added impacts of releasing GHGs higher in the atmosphere).

The other dramatic factor about the ICT industry is the rate of innovation. Areas that were in their infancy a few years ago can suddenly emerge as very significant factors in the industry, e.g., consider blockchain, which has emerged from nowhere to add around 0.1% of global emissions. Bitcoin alone (one of the cryptocurrencies supported by blockchain) has a carbon footprint equivalent to the whole of Switzerland.

**Figure 1:**
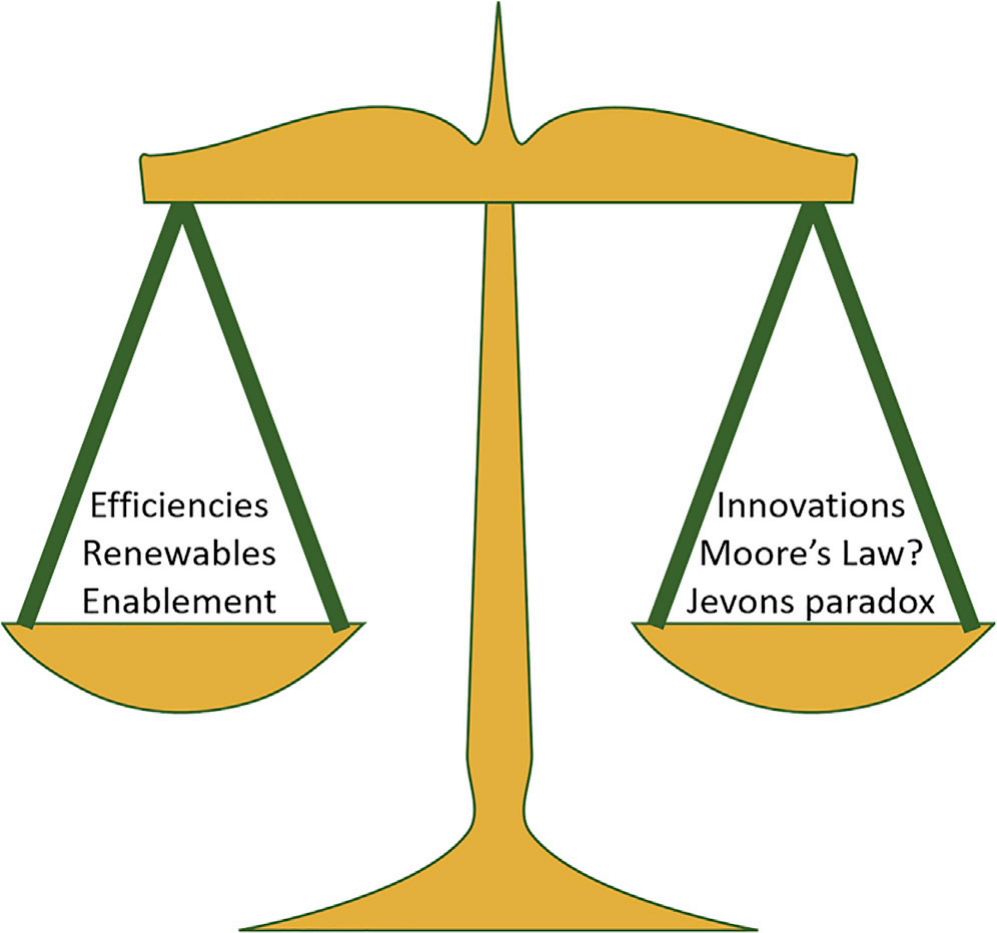
Competing Factors in the Future Carbon Emissions of ICT On the left, we have several factors helping to reduce ICT emissions, starting with efficiency gains. For example, Moore’s Law has been a huge factor in achieving efficiencies since the advent of computing (Moore’s Law states that the number of transistors in an integrated circuit doubles every two years, leading to energy efficiencies). Complementing this, many sectors of the ICT industry are increasing the percentage of energy from renewables, with big strides being made in data centers but more difficult to achieve in the decentralized Internet. There are also important enablement arguments, which claim that advances in ICT result in lower emissions in other sectors; for example, video-conferencing reduces the need to travel—a significant factor during the current COVID-19 pandemic. On the opposite side, many observers argue that the period governed by Moore’s Law is coming to an end. In addition, the effect of the Jevons Paradox is often overlooked. Empirical evidence has shown that in spite of 7 decades of energy efficiencies in ICT, the carbon footprint has steadily risen. This is a classic example of the paradox, whereby efficiency gains are swallowed up by increasing demand, e.g., by saving money from lower energy bills, there is an inclination to use this money elsewhere. This can apply within a sector (“local” Jevons) or across sectors (“global” Jevons), with the latter potentially reversing enablement gains. Finally, there is the large-scale investment and fast development of what are potentially power-hungry areas of innovation, including the Internet of Things, which will massively expand the number of devices worldwide, data centers and cloud computing fueled by the thirst for big data, and AI techniques used to analyze this big data, not forgetting the previously mentioned expansion in the use of blockchain. It is as yet unclear how this will unfold, but what is clear is that there is a significant risk that without intervention and/or regulation, the emissions associated with ICT could increase significantly at a time when they need to decrease. I finish with one graph produced by from our study (see Figure 2). This takes the most optimistic view of technology going forward from the various predictions considered in our study, assuming that GHG emissions remain stable at 2020 levels. The key point from this diagram is that, even with this optimistic projection, this is nowhere near enough to meet the Paris targets of achieving a 1.5°C warming, with the relative share of global emissions from ICT rising to more than a third of all emissions.

But what of the future? Some researchers claim that the GHG emissions of ICT are starting to flatten out due to major efficiency gains in the sector, particularly for data centers. While this is partially true, e.g., with significant efficiency gains in hyperscale data centers, the broader picture is more nuanced, with various competing factors as visualized in Figure 1.

**Figure 2:**
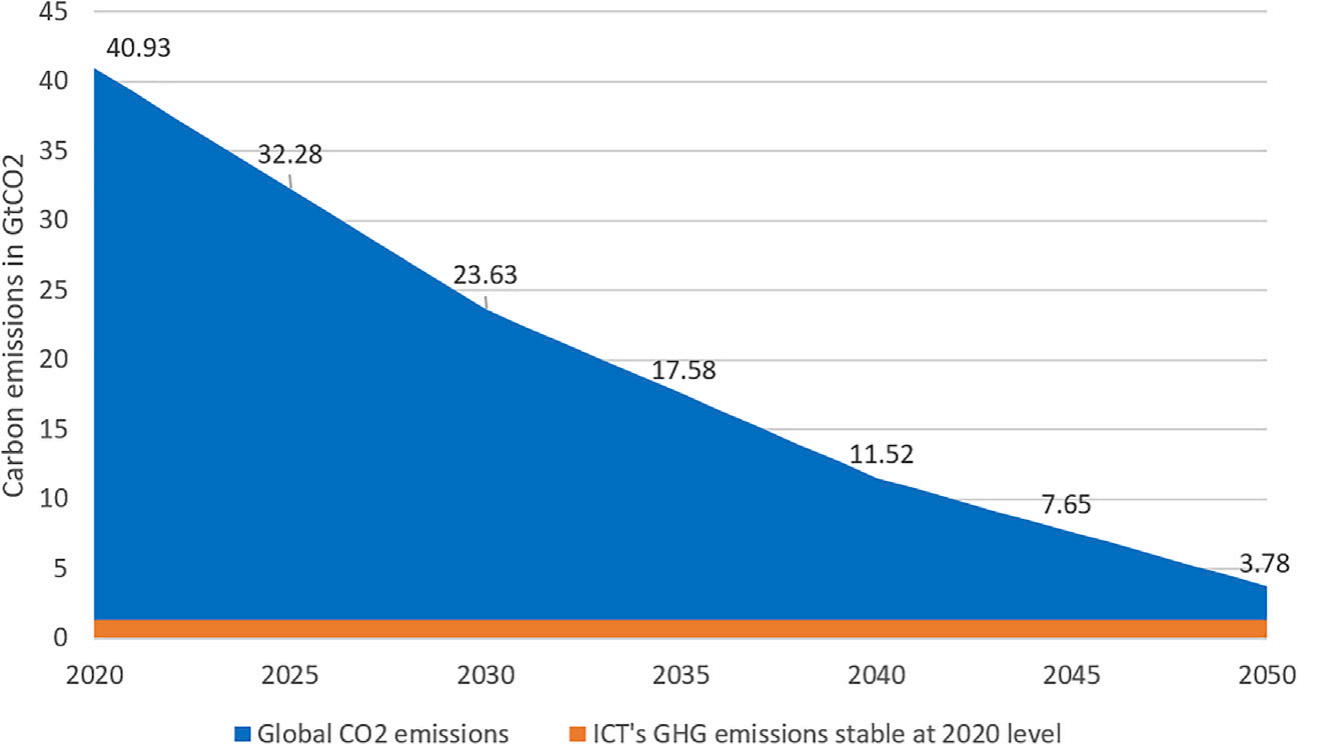
ICT’s Share of Carbon Emissions against Paris Targets Assuming a Stable State ICT’s GHG emissions, with an optimistic projection that it remains stable until 2050, and global CO_2_ emissions reduced in line with 1.5°C under scenario SSP2-19. Labels indicate ICT’s share of global CO_2_ emissions in percent.

### Final Remarks

So how does this end? How do we resolve these two very different perspectives of technology going forward? The simple answer is that nobody knows, but our current times give cause for optimism. This opinion piece was written during the COVID-19 period, and this has given us a glimpse of nature recovering and the inherent resilience in nature that allows it to recover in spite of the anthropogenic impacts we have imposed. There is equally a belief that when we come out of this period, things must change, and we should build back better. There is a future where technology underpins a more sustainable society (and not just referring to environmental sustainability but all facets covered by the sustainability triple bottom line; that is, environmental, economic and social sustainability), and there is a future where technology sits in harmony with nature. That is a future well worth working toward.

Returning to Dickens, it is intriguing to see how he finished his masterpiece. At one level, the book ends in tragedy with chaos all around and the major character, Sydney Carton, being beheaded. But the ending is actually one of hope and some would say resurrection as Dickens overlays the imaginary last words of Sydney Carton to include the well-known last line, “It is a far, far better thing that I do, than I have ever done; it is a far, far better rest that I go to than I have ever known.”
